# An Italian functional genomic resource for *Medicago truncatula*

**DOI:** 10.1186/1756-0500-1-129

**Published:** 2008-12-15

**Authors:** Andrea Porceddu, Francesco Panara, Ornella Calderini, Lorna Molinari, Paola Taviani, Luisa Lanfaloni, Carla Scotti, Maria Carelli, Laura Scaramelli, Gianluca Bruschi, Viviane Cosson, Pascal Ratet, Henri de Larembergue, Gerard Duc, Efisio Piano, Sergio Arcioni

**Affiliations:** 1 CNR Istituto di Genetica Vegetale Perugia, via Madonna Alta, 130 Perugia, Italy; 2 Dipartimento di Biologia Cellulare e Ambientale, Università degli Studi di Perugia, Via Pascoli 06123 Perugia, Italy; 3 CRA-FLC Centro per le Produzioni Foraggere e Lattiero-casearie, Viale Piacenza 29 –, 26900 Lodi, Italy; 4 Institut des Sciences du Végétal CNRS bat 23 – Avenue de la Terrasse –, 91198 Gif-sur-Yvette, Cedex –, France; 5 INRA URLEG Dijon BP86510, 21065 Dijon cedex, France; 6 Dipartimento di Scienze Agronomiche e Genetica Vegetale Agraria, Università degli Studi di Sassari, via E. de Nicola, 07100 Sassari, Italy

## Abstract

**Background:**

*Medicago truncatula *is a model species for legumes. Its functional genomics have been considerably boosted in recent years due to initiatives based both in Europe and US. Collections of mutants are becoming increasingly available and this will help unravel the genetic control of important traits for many species of legumes.

**Findings:**

Our report is on the production of three complementary mutant collections of the model species *Medicago truncatula *produced in Italy in the frame of a national genomic initiative. Well established strategies were used: *Tnt1 *mutagenesis, TILLING and activation tagging. Both forward and reverse genetics screenings proved the efficiency of the mutagenesis approaches adopted, enabling the isolation of interesting mutants which are in course of characterization. We anticipate that the reported collections will be complementary to the recently established functional genomics tools developed for *Medicago truncatula *both in Europe and in the United States.

## Background

*Medicago truncatula *has emerged as one of the two model systems for legume species.

The *Medicago truncatula *consortium (supported by US National Science Foundation and Samuel Roberts Noble Foundation in the USA, and in Europe, mainly by the European Union) has made significant achievements in genome and EST sequencing with a goal of completion of the gene space in 2008 [1]. The amount of information gained from the sequencing will assist studies related to gene function discovery. At the moment three complementary strategies have been chosen to create large mutant collections in *M. truncatula*: transposon tagging, fast neutron mutagenesis and TILLING. T-DNA tagging, one of the most popular strategies in *Arabidopsis*, did not represent the best option for *M. truncatula *because of the lack of a high throughput transformation system [2,3]. A population of more than 7600 *Tnt1 *lines was recently published [4] and is being developed as a public resource at the Samuel Roberts Noble Foundation . Functional genomics platforms are also available in Europe at the John Innes Genome Laboratory  which, at the moment, provides access to a large population of deletion tilled lines and tilled lines for reverse genetic screening. Both resources were established during the course of the project "GLIP": Grain Legumes Integrated Project, funded by the European Union . In addition a large *Tnt1 *collection (approximately 8000 plants) was established in the Jemalong 2HA background during the same project [5]. In the present paper we will report on three *M. truncatula *mutant collections established in Italy and of their potential benefits to the international scientific community for functional genomic studies. The collections were produced in the frame of the Italian functional genomics project "Post-Genomics of Forage Legumes RBNE018BHE" sponsored by the Italian Ministry of University and Research-Funds for Basic Research (MUR/FIRB).

## Findings

### A *Tnt1 *mutant collection

We have regenerated approximately 1000 *Tnt1 *mutant lines following current protocols with minor modifications [6]. A starter line from the R108-1 genotype harbouring 3 *Tnt1 *insertions was produced via *Agrobacterium *transformation of the construct Tnk23 [6]; the starter line was used as a source of leaf explants in 6 regeneration experiments. Among the 950 R0 lines producing seed, a random sample of 40 lines from different regeneration events was evaluated for transposition by Southern analysis. The average number of new insertion events was estimated to be 15–20. Considering the average number of insertions and the total number of lines available, the *Tnt1 *mutant collection produced represents a sum of approximately 14000–19000 insertions in the *M. truncatula *genome. To evaluate the ability of *Tnt1 *to target gene-rich regions as reported in the literature [4,7] we recovered *Tnt1 *FSTs via transposon display protocols [8]. In a small scale experiment 96 FSTs were recovered from 16 plants. BLAST analysis of those FSTs showed that at least 47% of the insertions are within genes, 27% are in sequenced but not yet annotated *Medicago truncatula *BAC clones and 25% do not show similarity with any sequence in the database [see Additional File 1]. This preliminary molecular analysis of the *Tnt1 *insertion sites confirms the data from Tadege et al., 2008 [4]; in fact in a larger scale experiment these authors reported an overall FST match with *Medicago truncatula *sequences in the database of 78.6%, 60.2% of which had high homology with a range of known genes.

To assess the potential of the mutant collection, forward genetic screens were carried out to identify visible mutant phenotypes. On a relatively large subset of the collection (approx. 600 progenies) the percentage of putative mutants observed were as follows: 4% shoot morphology, 8.5% root morphology, 4% nodules. In addition, genomic DNA was extracted from all the plants of the collection and was arranged for reverse genetic screenings. Because the genome coverage of our *Tnt1 *population is relatively small compared to other resources we suggest our population as a complement and integration to those already available for reverse genetics screening [4,5].

**Figure 1:**
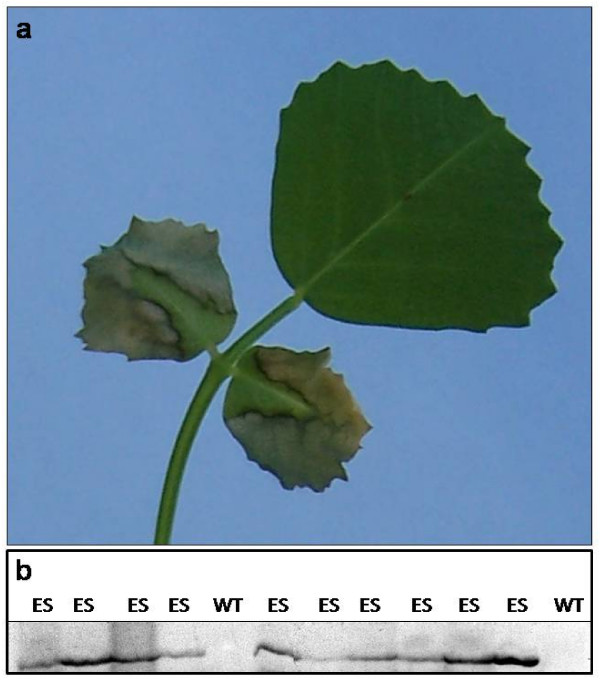
**a) Leaves of the mutant plant C43 showing symptoms of early senescence**. b) retrotransposon display analysis of 12 segregating progenies of C43: the band present only in the early senescing plants (ES) was chosen as a candidate gene; (WT = wild type)

In depth analysis of a few mutants selected from the collection is in progress. As an example, a mutant showing premature leaf death and loss is reported in Fig. 1. The mutation is inherited as dominant. A preliminary segregation analysis based on 10 FSTs recovered from the mutant DNA revealed a candidate gene responsible for the mutation which is a putative component of the RNA interference machinery (Calderini et al, unpubl.).

### An activation tagging sample collection

We report on the production of a small population of activation tagging lines produced as a test of transformation efficiency, which, in spite of its very modest size, allowed us to identify an interesting mutant. The population consists of 128 lines obtained via *Agrobacterium *transformation of R108-1 genotype with the pSKI074 vector [9] kindly provided by D. Weigel (MPI for Developmental Biology, Tubingen, Germany).

T1 progenies of the collection were screened for the presence of haemolytic saponins in the leaves using the assay reported in [10]. A mutant plant lacking haemolytic saponins in the leaves was identified. The mutation resulted in the loss-of function of a novel member of the cytochrome P450 family; co-segregation analysis proved that the phenotype is dependent on the mutation identified; an extensive characterization of this mutant is underway (Scotti et al, unpubl.). The involvement of cytochrome P450 in saponin biosynthesis was recently demonstrated in oat roots [11]; we believe that the mutation reported here will help to clarify the biochemical pathway leading to saponin synthesis in *Medicago truncatula *leaves and it will open possibilities for novel manipulations of such compounds.

### A TILLING collection

*M. truncatula *seeds from cv. Jemalong genotype 2HA10-9-3 were treated with 0.15% EMS [12] to generate a mutant collection. The genotype 2HA10-9-3 was chosen by the European Union as the background of the large scale *Tnt1 *insertional mutagenesis carried out during the Grain Legumes Integrated Project because of its higher regeneration ability compared to the genotype Jemalong A17 and of its close genetic similarity to the sequenced genotype A17 [13]. Our TILLING population was therefore created in a genetic background that could contribute allelic series for the EU *Tnt1 *functional genomics platform, whilst also being useful for other genetic backgrounds. The EMS treatment induces numerous point mutations and the generation of these mutants does not require extensive tissue culture work as is the case for the strategies previously reported (*Tnt1 *and T-DNA tagging). About 2500 M1 plants were grown to produce the M2 generation. M2 seeds were collected from 2281 M1 individuals together with 65 tester plants from the control treatment (0% EMS).

**Figure 2:**
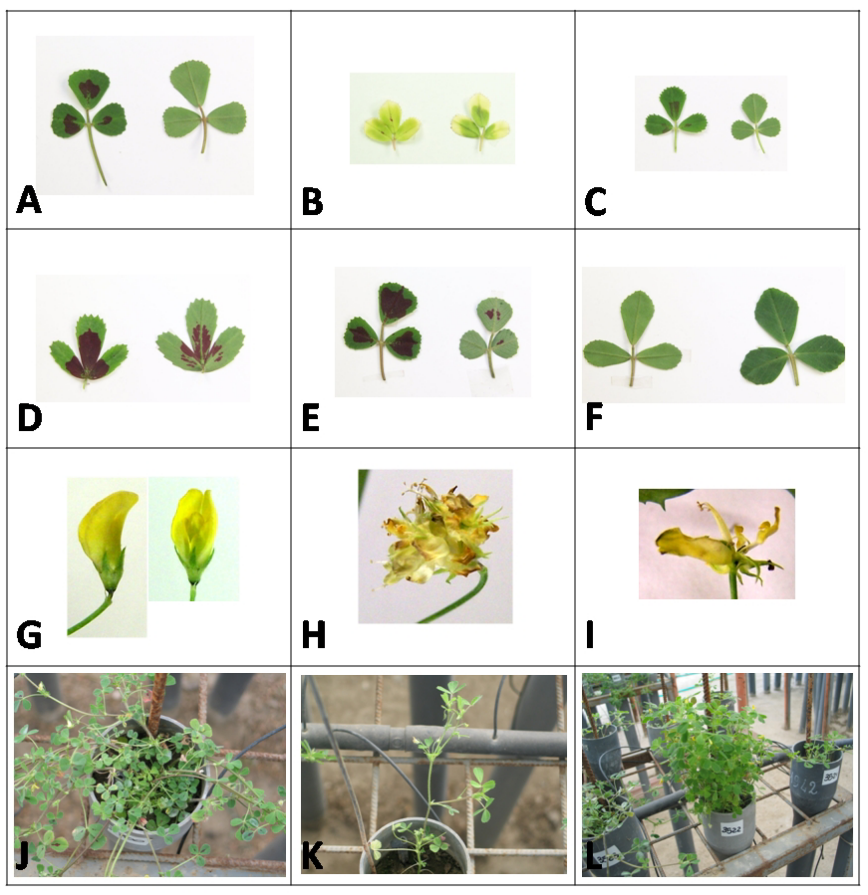
**TILLING collection: a selection of morphological mutants**. A-F) leaves of wild type (A) *M. truncatula *and of mutant (B-F) plants showing changes in pigmentation (B), size (C), morphology (D), size of the red spot (E, F). In plates A-E the leaf adaxial side is shown on the left, the abaxial side is shown on the right, in plate F the leaf adaxial side is shown on the right, the abaxial side is shown on the left. G-I) Flowers of wild type plants (G) and of different mutant plants (H, I) with abnormal floral organs. J-L growth habit of wild type plants (J) and of mutant plants (K: no lateral shoots; L: erect stems).

The M2 generation (1658 families, represented by 1–5 plants/family for a total of 2560 plants) and 34 tester families were grown in a cold greenhouse and phenotypically screened to identify mutants (Table 1, Fig. 2). DNA was extracted from almost all the M2 families and M3 seed has been collected; about 2000 plants are present in the final EMS mutant collection (DNA and M3 seed). DNA samples of equal amount from eight and twelve individual plants were pooled in such a way that each individual was randomly included in both types of bulks; this procedure allows the unambiguous identification of the individual plant carrying the mutation.

**Table 1:** Phenotypic classes observed in the screening of the TILLING collection.

**Phenotypic class**	**Number of**	**Frequency**
	**mutated plants**	**(%)**

**Seedling stage**		

Albino	30	0.40

Chlorotic	48	0.64

Pale green	34	0.43

Short and compact root	62	0.87

Deformed root (growth-arrested)	84	1.26

Arrested embryos	781	12.3

**Adult plant stage**		

**Plant morphology**		

Stunted growth	145	5.66

Very stunted growth	27	1.05

Extreme dwarf, deformed shoots	2	0.08

Extreme dwarf	41	1.6

Extreme short internode	4	0.16

Short internode	4	0.16

Erect stem	1	0.04

Curled stem	2	0.08

**Leaf morphology**		

Small leaves	15	0.58

Abnormal leaf senescence	4	0.16

Deformed leaves	24	0.94

Partially unifoliate leaves	1	0.04

**Pigmentation**		

Purple leaves	1	0.04

Pale green leaves	4	0.16

No leaf mark	9	0.35

7229 seeds for the seedling stage and 2560 plants for the adult stage were screened.

The validation of the mutagenic treatment was performed by screening the collection for three genes of interest by the TILLING technique, in collaboration with the Genomic Platform (PGP) of the Parco Tecnologico Padano (Lodi, Italy). The following genes were considered for screening: 1) the cytochrome P450 related to the absence of haemolytic saponins, 2) the *M. truncatula *phytase gene *Mt*PHY1 [14], and the putative orthologue of the trypsin inhibitor gene MsTI from *M. scutellata *[15] which was identified by BLASTn searches in GenBank and TIGR *M. truncatula *Gene Index databases. The amplicons from the putative mutant individuals and from wild type plants were sequenced and compared to analyse single base differences (Table 2).

**Table 2:** TILLING analysis for MtPHY1 and the putative *M. truncatula *orthologue of MsTI from *Medicago scutellata*

Gene	GenBank	Primer pair	No. of mutations	Type of mutation
MtPHY1	AY878355	5'-AACAATGCCGGTTTCAGGTC 5'-TTGAAGGCGCAGAAACCTCT	8	C → T (4) G → A (4)

Trypsin inhibitor	AC135311	5'-TGTTTGTTTCGTCTGGAGCA 5'-GTGGACGGTCTCCCAGACTC	7	C → T (2) G → A (5)

Different point mutations were found: G to A transitions (60%) and C to T transitions (40%) (Tab. 2). The estimated rate of mutation was 1 mutation/Kbp/400 plants, similar to the collection generated by the R. Cook laboratory [16]. In particular for MtPHY1 and the *M. truncatula *MsTI genes we were able to recover three and six mutants respectively bearing amino acid substitutions. The M3 progenies of the mutant individuals for the three genes of interest are currently subject to phenotypic screening. Moreover 4 alleles were recovered for the cytochrome P450 gene related to the absence of haemolytic saponin phenotype. Three of the mutants recovered carry amino acid substitutions, and one mutant carries a premature stop codon. This allelic series is currently used for genetic complementation tests; two of these mutants were analysed and they lack haemolytic saponins in the leaves in agreement with the role of the reported gene in the pathway. The characterization of the two EMS alleles together with the previously reported loss-of-function mutant underlines the usefulness of the current functional genomics approaches. In-depth analysis of the allelic series regarding the saponin-deficient mutants will be a matter for future publication (Scotti et al., unpub). The TILLING collection reported in the present paper is therefore a valuable tool for the complementation analysis of mutants isolated with other strategies.

## Conclusion

We have produced different functional genomic tools for *Medicago truncatula *that have proved efficient for both forward and reverse genetics in this species. We anticipate the possibility that the reported collections will be of public relevance because they will be accessible to the scientific community. The TILLING and the *Tnt1 *collections are publicly available for screening via reverse genetics on a cost recovery basis. Forward genetic screenings are ongoing for all the mentioned collections; interested researchers are welcome to enquire in order to organize visits at the host Institutions (CNR-IGV Perugia, CRA-FLC Lodi). For both types of screenings interested scientists are encouraged to contact the corresponding author. At the moment a web site for the reported collections is under construction at . In addition extensive sequencing of FSTs from the *Tnt1 *collection would be highly desirable also in the frame of future collaborative efforts at the international level. All the sequencing information could be integrated in a public database hosting data of various kinds for the mutants, such as already developed to a high standard for *Arabidopsis*. Public stock centres such as TAIR and NASC which serve *Arabidopsis *would be extremely valuable for the *Medicago *research community. We expect to be able to contribute our collections to such facilities once organized.

## Abbreviations

EST: Expressed Sequence Tag; FST: Flanking Sequence Tag; TILLING: Targeting Local Lesions IN Genomes; EMS: Ethyl-Methane Sulfonate.

## Competing interests

The authors declare that they have no competing interests.

## Authors' contributions

AP, FP, LM, PT and OC contributed in plant regeneration and molecular analysis of the *Tnt1 *collection; OC wrote the paper, AP and SA conceived experiments; AP and LL performed EMS treatment and produced M1 and part of M2 seeds; CS, MC, LS and GB grew the M1 and M2 generations, produced DNA bulks and performed molecular analysis of the activation tagging sample collection; VC, PR, HdL and GD performed the phenotypic screening in the *Tnt1 *collection, EP supervised the work on the TILLING collection.

All authors read and approved the final manuscript.

## Supplementary Material

Additional file 1 **Blast analysis of 96 FSTs from 16 *Tnt1 *mutants of *Medicago truncatula *R-108**. The data provided represent the Blast analysis of 96 FSTs recovered from 16 plants of *Medicago truncatula *R-108 harbouring the *Tnt1 *transposon. Click here for file
