# Supplementary material for: An Italian functional genomic resource for Medicago truncatula
Source: BMC Res Notes. 2008 Dec 15;1:129. doi: 10.1186/1756-0500-1-129 (PMC2633015; doi:10.1186/1756-0500-1-129)
Supplement: Additional file 1 — Blast analysis of 96 FSTs from 16 Tnt1 mutants of Medicago truncatula R-108. The data provided represent the Blast analysis of 96 FSTs recovered from 16 plants of Medicago truncatula R-108 harbouring the Tnt1 transposon. [file 1756-0500-1-129-S1.doc]

**Supplemental Table 1**. Blast analysis of 96 FSTs from 16 Tnt1 mutants of *Medicago truncatula* R-108.

| **Sequence ID** | **Mutant line** | **Similarity** | **Blast analysis** | **Score** | **Classification** |
| --- | --- | --- | --- | --- | --- |
| 1 | 555 | gi|55667664|gb|AC149131.3| Medicago truncatula chromosome 7 clone mth2-19e15 | BLAST-N | 46.1 bits (23) | Mt BAC clone |
| 2 | 555 | gb|ABE93148.1| Leucine-rich repeat; Leucine-rich repeat, cysteine-containing [Medicago truncatula] | BLAST-X | 46.2 bits (108) | Putative coding region |
| 3 | 555 | gi|124360062|gb|ABN08078.1| Protein of unknown function DUF889, eukaryote [Medicago truncatula] | BLAST-X | 95.5 bits (236) | Putative coding region |
| 4 | 553 | gi|76559722|emb|CT009492.5| M.truncatula DNA sequence from clone MTH2-93A21 on chromosome 3 | BLAST-N | 67.9 bits (34) | Mt BAC clone |
| 5 | B1 | emb|CAN64227.1| hypothetical protein [Vitis vinifera] | BLAST-X | 111 bits (277) | Putative coding region |
| 6 | 554 | gi|87162935|gb|ABD28730.1| Ribonuclease H [Medicago truncatula] | BLAST-X | 45.4 bits (106) | Putative coding region |
| 7 | 550 | No significant similarity |  |  | No similarity |
| 8 | 546 | gi|126635092|emb|CU424490.2| Medicago truncatula chromosome 5 clone mth2-95h16 | BLAST-N | 103 bits (52) | Mt BAC clone |
| 9 | 546 | No significant similarity |  |  | No similarity |
| 10 | B1 | gb|AAD27882.2|AF139470_1 chlorophyll a/b-binding protein CP24 precursor [Vigna radiata] | BLAST-X | 82.8 bits (203) | Putative coding region |
| 11 | 546 | gi|121584080|gb|AC175685.3| Medicago truncatula chromosome 2 BAC clone mte1-55k6 | BLAST-N | 52.0 bits (26) | Mt BAC clone |
| 12 | 555 | No significant similarity |  |  | No similarity |
| 13 | 546 | gi|51872224|gb|AY596916.1| Viburnum sieboldii granule-bound starch synthase I (GBSSI) gene | BLAST-N | 46.1 bits (23) | Putative coding region |
| 14 | 555 | gi|89570868|emb|CR962125.2| Medicago truncatula chromosome 5 clone mth2-179a9 | BLAST-N | 58.0 bits (29) | Mt BAC clone |
| 15 | 546 | gi|87240518|gb|ABD32376.1| Lipolytic enzyme, G-D-S-L [Medicago truncatula] | BLAST-X | 68.2 bits (165) | Putative coding region |
| 16 | 555 | gi|28392990|gb|AAO41930.1| putative protein kinase [Arabidopsis thaliana] | BLAST-X | 76.6 bits (187) | Putative coding region |
| 17 | 546 | No significant similarity |  |  | No similarity |
| 18 | 550 | ref|XP_550188.1| unknown protein [Oryza sativa (japonica cultivar-group)] | BLAST-X | 48.5 bits (114) | Putative coding region |
| 19 | 558 | No significant similarity |  |  | No similarity |
| 20 | B7 | ref|NP_974329.1| rhomboid family protein / zinc finger protein-related [Arabidopsis thaliana] | BLAST-X | 159 bits (401) | Putative coding region |
| 21 | 554 | gi|156071563|gb|AC147363.33| Medicago truncatula clone mth2-17c13 | BLAST-N | 54.0 bits (27) | Mt BAC clone |
| 22 | 547 | No significant similarity |  |  | No similarity |
| 23 | 554 | gi|168239900|gb|AC171618.20| Medicago truncatula clone mth2-144n17 | BLAST-N | 254 bits (128) | Mt BAC clone |
| 24 | 547 | gi|157338867|emb|CAO42218.1| unnamed protein product [Vitis vinifera] | BLAST-X | 50.8 bits (120) | Putative coding region |
| 25 | 549 | No significant similarity |  |  | No similarity |
| 26 | 547 | gb|DQ285630.1| Oryza sativa (indica cultivar-group) putative nitrate-induced NOI protein (75-1-127BAC12.1) | BLAST-N | 42.1 bits (21) | Putative coding region |
| 27 | 553 | No significant similarity |  |  | No similarity |
| 28 | 546 | gi|147766034|emb|CAN70213.1| hypothetical protein [Vitis vinifera] | BLAST-X | 94.7 bits (234) | Putative coding region |
| 29 | B7 | gb|ABK94962.1| unknown [Populus trichocarpa] | BLAST-X | 154 bits (389) | Putative coding region |
| 30 | 547 | No significant similarity |  |  | No similarity |
| 31 | 546 | gi|124360445|gb|ABN08455.1| Leucine-rich repeat; Leucine-rich repeat, cysteine-containing [Medicago truncatula] | BLAST-X | 113 bits (283) | Putative coding region |
| 32 | 553 | No significant similarity |  |  | No similarity |
| 33 | 555 | gi|58418457|gb|AC144430.14| Medicago truncatula clone mth2-16j2 | BLAST-N | 301 bits (152) | Mt BAC clone |
| 34 | 549 | gi|157351928|emb|CAO42892.1| unnamed protein product [Vitis vinifera] | BLAST-X | 55.1 bits (131) | Putative coding region |
| 35 | 551 | gi|157350744|emb|CAO40835.1| unnamed protein product [Vitis vinifera] | BLAST-X | 40.8 bits (94) | Putative coding region |
| 36 | 553 | emb|CT967318.5| M.truncatula DNA sequence from clone MTH2-3P14 on chromosome 3 | BLAST-N | 73.4 bits (80) | Mt BAC clone |
| 37 | 555 | gb|AF047054.1|AF047054 Glycine max putative 3,4-dihydroxy-2-butanone kinase (dhbk) mRNA, partial cds | BLAST-N | 59.0 bits (64) | Putative coding region |
| 38 | 552 | gi|87240698|gb|ABD32556.1| hypothetical protein MtrDRAFT_AC149131g9v2 [Medicago truncatula] | BLAST-X | 98.2 bits (243) | Putative coding region |
| 39 | 558 | gi|119359657|emb|CU137656.2| Medicago truncatula chromosome 5 clone mte1-2l20 | BLAST-N | 77.8 bits (39) | Mt BAC clone |
| 40 | 560 | gi|169153923|emb|CAQ15302.1| novel protein similar to H.sapiens TBC1D10A, TBC1 domain family, member 10A (TBC1D10A) [Danio rerio] | BLAST-X | 41.6 bits (96) | Putative coding region |
| 41 | B1 | gb|AC136286.30| Medicago truncatula clone mth2-6c9 | BLAST-N | 89.7 bits (98) | Mt BAC clone |
| 42 | B1 | gb|AC142526.5| Medicago truncatula clone mth2-34h20 | BLAST-N | 48.1 bits (24) | Mt BAC clone |
| 43 | 550 | No significant similarity |  |  | No similarity |
| 44 | 549 | gb|ABE93445.1| hypothetical protein MtrDRAFT_AC123571g34v1 [Medicago truncatula] | BLAST-X | 62.0 bits (149) | Putative coding region |
| 45 | B7 | ref|NP_197091.1| glycosyl hydrolase family 81 protein [Arabidopsis thaliana] | BLAST-X | 46.6 bits (109) | Putative coding region |
| 46 | 558 | gi|12054977|emb|CAC20725.1| putative chalcone synthase [Medicago truncatula] | BLAST-X | 348 bits (892) | Putative coding region |
| 47 | 547 | No significant similarity |  |  | No similarity |
| 48 | B1 | No significant similarity |  |  | No similarity |
| 49 | 553 | No significant similarity |  |  | No similarity |
| 50 | 558 | gi|52782510|gb|AC134823.41| Medicago truncatula chromosome 6 clone mth2-20d18 | BLAST-N | 60.0 bits (30) | Mt BAC clone |
| 51 | 555 | gi|157329704|emb|CAO41596.1| unnamed protein product [Vitis vinifera] | BLAST-X | 46.2 bits (108) | Putative coding region |
| 52 | 560 | No significant similarity |  |  | No similarity |
| 53 | 558 | gi|52353642|gb|AAU44208.1| unknown protein [Oryza sativa (japonica cultivar-group)] | BLAST-X | 84.0 bits (206) | Putative coding region |
| 54 | 554 | gi|92885053|gb|ABE87609.1| Plant protein of unknown function [Medicago truncatula] | BLAST-X | 110 bits (274) | Putative coding region |
| 55 | 549 | No significant similarity |  |  | No similarity |
| 56 | 547 | No significant similarity |  |  | No similarity |
| 57 | 550 | gi|49744953|gb|AC148815.2| Medicago truncatula chromosome 2 clone mth2-13k15 | BLAST-N | 244 bits (123) | Mt BAC clone |
| 58 | 546 | gb|ABE81869.1| hypothetical protein MtrDRAFT_AC137702g10v2 [Medicago truncatula] | BLAST-X | 112 bits (279) | Putative coding region |
| 59 | 550 | gi|125524478|gb|EAY72592.1| hypothetical protein OsI_000439 [Oryza sativa (indica cultivar-group)] | BLAST-X | 51.6 bits (122) | Putative coding region |
| 60 | 560 | gi|62909796|emb|CR954196.1| Medicago truncatula chromosome 5 clone mth2-157e5 | BLAST-N | 311 bits (157) | Mt BAC clone |
| 61 | B21 | emb|CAC20725.1| putative chalcone synthase [Medicago truncatula] | BLAST-X | 348 bits (892) | Putative coding region |
| 62 | B21 | gb|AY372416.1| Medicago truncatula HCR4 gene | BLAST-N | 100 bits (110) | Putative coding region |
| 63 | 561 | No significant similarity |  |  | No similarity |
| 64 | 555 | No significant similarity |  |  | No similarity |
| 65 | 555 | gi|157347439|emb|CAO18076.1| unnamed protein product [Vitis vinifera] | BLAST-X | 91.7 bits (226) | Putative coding region |
| 66 | 561 | gi|114649663|emb|CU137662.1| Medicago truncatula chromosome 5 clone mth2-155l8 | BLAST-N | 569 bits (287) | Mt BAC clone |
| 67 | 547 | gi|157358290|emb|CAO65927.1|unnamed protein product [Vitis vinifera] | BLAST-X | 181 bits (459) | Putative coding sequence |
| 68 | 558 | gi|190714797|emb|CU651567.7|M.truncatula DNA sequence from clone MTE1-64C6 on chromosome 3 | BLAST-N | 137 bits (69) | Mt BAC clone |
| 69 | 560 | No significant similarity |  |  | No similarity |
| 70 | 558 | gi|90656458|gb|DQ455511.1|Medicago truncatula cDNA-AFLP fragment BT42M31_461 | BLAST-N | 67.9 bits (34) | Putative coding region |
| 71 | 558 | gi|157330166|emb|CAO44616.1|unnamed protein product [Vitis vinifera] | BLAST-X | 93.6 bits (231) | Putative coding region |
| 72 | 547 | gi|157353065|emb|CAO44955.1|unnamed protein product [Vitis vinifera] | BLAST-X | 75.5 bits (184) | Putative coding region |
| 73 | 555 | No significant similarity |  |  | No similarity |
| 74 | 561 | gi|15232718|ref|NP_187569.1|amine oxidase family protein [Arabidopsis thaliana] | BLAST-X | 181 bits (460) | Putative coding region |
| 75 | 558 | No significant similarity |  |  | No similarity |
| 76 | 546 | gi|144225838|emb|CU326391.2|Medicago truncatula chromosome 5 clone mth2-182c4 | BLAST-N | 305 bits (154) | Mt BAC clone |
| 77 | 550 | gi|111186006|gb|AC147713.21|Medicago truncatula clone mth2-138b11 | BLAST-N | 61.9 bits (31) | Mt BAC clone |
| 78 | 546 | No significant similarity |  |  | No similarity |
| 79 | 555 | No significant similarity |  |  | No similarity |
| 80 | 558 | gi|189458710|emb|CU469563.2|Medicago truncatula chromosome 5 clone mth4-41k12 | BLAST-N | 1181 bits (596) | Mt BAC clone |
| 81 | 550 | No significant similarity |  |  | No similarity |
| 82 | 546 | gi|124361231|gb|AC195566.2| Medicago truncatula chromosome 2 BAC clone mth2-175g13 | BLAST-N | 178 bits (90) | Mt BAC clone |
| 83 | 562 | gb|ABN06084.1| Polynucleotidyl transferase, Ribonuclease H fold [Medicago truncatula] | BLAST-X | 127 bits (319) | Putative coding region |
| 84 | 558 | gi|118485196|gb|ABK94459.1| unknown [Populus trichocarpa] | BLAST-X | 76.3 bits (186) | Putative coding region |
| 85 | B7 | emb|CAO61249.1| unnamed protein product [Vitis vinifera] | BLAST-X | 313 bits (802) | Putative coding region |
| 86 | 562 | gb|ABN09154.1| RNA-directed DNA polymerase (Reverse transcriptase) [Medicago truncatula] | BLAST-X | 65.9 bits (159) | Putative coding region |
| 87 | 562 | emb|CAO48286.1| unnamed protein product [Vitis vinifera] | BLAST-X | 51.2 bits (121) | Putative coding region |
| 88 | 562 | emb|CAO14388.1| unnamed protein product [Vitis vinifera] | BLAST-X | 178 bits (451) | Putative coding region |
| 89 | 562 | gb|ABW81103.1| unknown [Cleome spinosa] | BLAST-X | 70.5 bits (171) | Putative coding region |
| 90 | 562 | gb|AC146561.22| Medicago truncatula clone mth2-12j18 | BLAST-N | 634 bits (702) | Mt BAC clone |
| 91 | 562 | emb|CT573053.1| Medicago truncatula chromosome 5 clone mte1-8e5 | BLAST-N | 792 bits (878) | Mt BAC clone |
| 92 | 562 | ref|YP_001389432.1| hypothetical protein CLI_0124 [Clostridium botulinum F str. Langeland] | BLAST-X | 43.5 bits (101) | Putative coding region |
| 93 | 555 | gi|157743440|gb|AC165429.3| Medicago truncatula chromosome 7 BAC clone mth2-59p23 | BLAST-N | 48.1 bits (24) | Mt BAC clone |
| 94 | 562 | gb|AC192957.2| Medicago truncatula chromosome 7 BAC clone mte1-32c12 | BLAST-N | 69.8 bits (76) | Mt BAC clone |
| 95 | 555 | gi|157335237|emb|CAO61067.1| unnamed protein product [Vitis vinifera] | BLAST-X | 82.4 bits (202) | Putative coding region |
| 96 | 546 | gi|51315499|gb|AC135798.31| Medicago truncatula clone mth2-31c16 | BLAST-N | 50.1 bits (25) | Mt BAC clone |
